# Recurrence of spontaneous pneumothorax six years after VATS pleurectomy: evidence for formation of neopleura

**DOI:** 10.1186/s13019-020-01233-9

**Published:** 2020-07-28

**Authors:** Peter Germonpre, Elke Van Renterghem, Nicolas Dechamps, Thierry Onghena, Joke Van Aken

**Affiliations:** 1 Centre for Hyperbaric Oxygen Therapy, Military Hospital Brussels, 1120 Brussels, Belgium; 2 Emergency Department, St Lucas Hospital, Ghent, Belgium; 3 Laerdal Medical Benelux, Vilvoorde, Belgium; 4 Thoracic Surgery Department, St Lucas Hospital, Ghent, Belgium; 5 Pathology Department, St Lucas Hospital, Ghent, Belgium

**Keywords:** Primary spontaneous Pneumothorax, Pleurectomy, Recurrence, VATS, Pleural regeneration

## Abstract

**Background:**

Primary Spontaneous Pneumothorax (PSP) is considered an absolute and definitive contraindication for scuba diving and professional flying, unless bilateral surgical pleurectomy is performed. Only then is there a sufficiently low risk of recurrence to allow a waiver for flying and/or diving.

**Case presentation:**

A young fit male patient who suffered a PSP 6 years ago, and underwent an uncomplicated videoscopic surgical pleurectomy, presented with a complete collapse of the lung on the initial PSP side. Microscopic examination of biopsies showed a slightly inflamed tissue but otherwise normal mesothelial cells, compatible with newly formed pleura.

**Conclusions:**

Even with pleurectomy, in this patient, residual mesothelial cells seem to have had the capacity to create a completely new pleura and pleural space. The most appropriate surgical technique for prevention of PSP may still be debated.

## Background

Primary Spontaneous Pneumothorax (PSP) is considered an absolute and definitive contra-indication for scuba diving [1]. An exception could be made if bilateral surgical pleurectomy is performed, with resection of any visible bullae or blebs, and then only if no further structural or functional abnormalities are found (on high-resolution CT scan and extensive pulmonary function testing) [1–3]. Video assisted surgical pleurectomy (Video Assisted Thoracic Surgery - VATS) is nowadays considered the treatment of choice for these patients, as it offers a very low risk of recurrence for acceptable surgical morbidity [4, 5]. It is thought that surgical pleurectomy, with removal of most of the parietal pleura by sharp dissection and forceps peeling, has a lower recurrence rate than simple abrasion of the parietal pleural surface, as in the former, all pleura is stripped from the thoracic wall, producing fibrous scar tissue which completely obliterates the pleural cavity [6–8]. However, owing to the invasive nature of this procedure (which must be done bilaterally), not many divers take this step; therefore, follow-up data for divers is very scarce. Nevertheless, if this intervention were found to fail in its primary purpose, the current recommendations may have to be revised.

We present a case of ipsilateral recurrence of PSP in a young male, 6 years after the initial event, which was treated with VATS pleurectomy.

## Case presentation

A 31-year old, fit, healthy male, an occasional smoker, suffered acute severe right thorax pain during a bicycle tour. Clinical examination and chest X-ray revealed an important right-sided pneumothorax, which was treated with single-needed aspiration and was fully deployed after aspiration of 510 ml. However, a control X-ray after 36 h revealed a full recurrence with on expiration film a slight deviation of the trachea and upper mediastinum. Therefore, and because many of the sports activities the patient participated in involved atmospheric pressure changes (skiing, diving, flying), it was decided to proceed to surgical pleurectomy. This was performed with a classic three-channel VATS, in the 6th and 8th intercostal space. The parietal pleura was surgically stripped from the first rib, along the sympathetic nerve chain, to the diaphragm and along the internal mammary artery. No blebs or bullae were visualized. The suction drain was clamped on the 5th postoperative day, but because of a small apical loosening, suction was resumed for another 5 days. At that point, a control X-ray was normal and the suction drain was removed at day 10. The patient was able to fully resume working activities after 1 month. Pulmonary function tests were performed after 3 months and showed normal values (Vital Capacity 110%, FEV1 at 115%, DLCO 90% and Transfer Coefficient (DLCO/Alveolar Volume) of 99%.

Six years later, at rest, he again experienced some discomfort in the right chest cavity, which manifested mostly as dyspnea and some physical limitation during running. In retrospect, this chest discomfort may have started after having attended a pop concert a few days earlier. After a few days (because of the weekend) he sought a medical consultation and a chest X-ray again showed a complete collapse of the right lung.

**Fig. 1:**
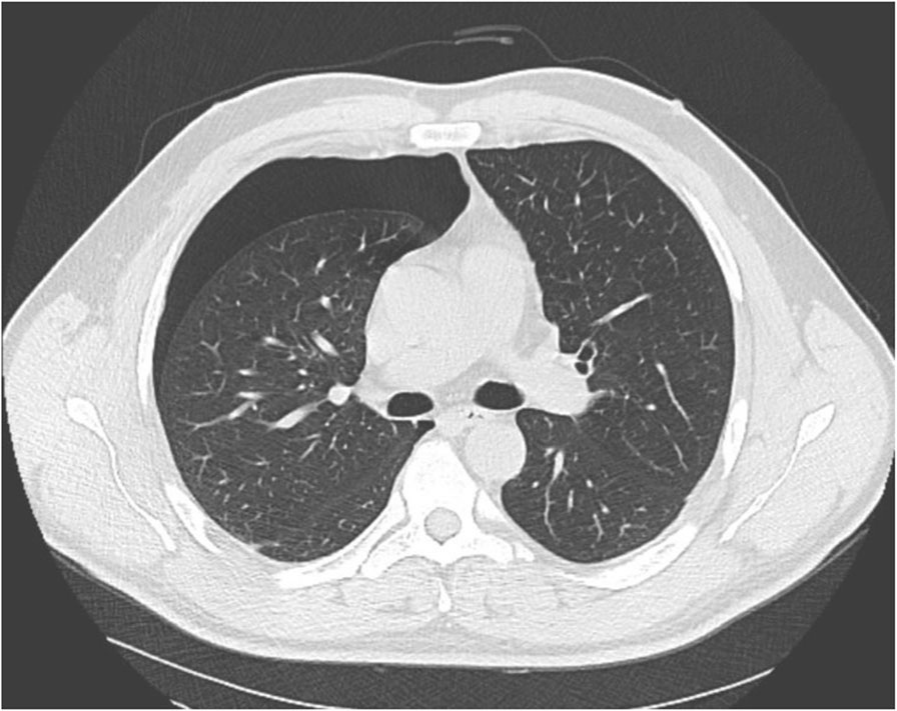
Recurrence of pneumothorax 6 years after VATS pleurectomy

A CT-scan confirmed a complete right-sided pneumothorax, with no adhesions in the pleural space, compression of the right lung and deviation of the mediastinum towards the left. A small subpleural bulla was identified in the superior and inferior lobes (Fig. 1).

A new thoracoscopic intervention was performed, with resection of the right apex, and prelevation of biopsies, prior to complete parietal surface scarring and chemical (talcum powder) pleurodesis. The postoperative course was uneventful. Examination of the biopsies showed the presence of reactive hyperplastic mesothelial cells on the parietal side, compatible with neo-pleura.

## Discussion

Primary Spontaneous Pneumothorax is defined as collapse of the lung without obvious external trauma. It is most frequently observed in young male patients, mostly smokers, and is thought to result from structural abnormalities in the lung or pleural tissue, with or without radiological or visual evidence of pulmonary blebs or bullae [4]. The risk of recurrence is high, either ipsilateral (16–52% within the first year) or contralateral (12.5%) [9]. Therefore, a simple needle aspiration or chemical pleurodesis are considered insufficient to provide adequate protection should the individual be involved with critical activities involving environmental pressure changes. As an example, should a pneumothorax occur during scuba diving at a depth of only 10 m, the expansion of the extrapulmonary air during ascent to the surface would entail a doubling of its volume (Boyle’s Law), possibly provoking a severe and life-threatening tension pneumothorax. Similarly, professional (military) pilots may be prohibited from resuming their duties after PSP unless a more definite (surgical) procedure has been performed [10–12]. However, it is recognized that while the risk is sufficiently low to allow resumption of these activities (estimated at 0.5%), it is still somewhat higher than in the reference population. Estimation of this risk is rendered difficult by the variable terminology used to describe the procedure. For example, the thoracoscopic administration of talcum powder or tetracycline is technically a chemical pleurodesis administered in a surgical way, so may be called ‘surgical pleurodesis’; however, the same name is given to a mechanical abrasion of the parietal pleura through thoracoscopy, as well as to VATS pleurectomy, which is a partial removal of the parietal pleura by means of incision and forceps. From the current literature, it is virtually impossible to distinguish the recurrence rates for each of these three interventions (or more if combinations are used) [13, 14].

Chemical or mechanical pleurodesis provokes an inflammatory state in the pleura, inducing the formation of adhesions, also because of fibronectin formation by injured mesothelial cells. After a period of 3 to 4 weeks, fibrosis of the pleural cavity occurs [15].

By surgically removing the parietal pleura, a raw, bleeding surface is created which subsequently heals by scarring with complete removal of parietal pleura structures and this is the main reason why, in divers and aviators, pleurectomy would be preferred over chemical pleurodesis or scarring of the parietal pleura. This would allow the pleural space to be completely obliterated without a possibility for re-creation of a virtual pleural space [7, 16]. In nature, only one species of mammal, the elephant, has evolved such a fibrous structure in lieu of a ‘virtual space’ pleura, allowing them to use their trunk as a snorkel, and breathe at much higher negative pressures than is possible in humans. Elephants cannot develop pneumothoraxes [17].

**Fig. 2:**
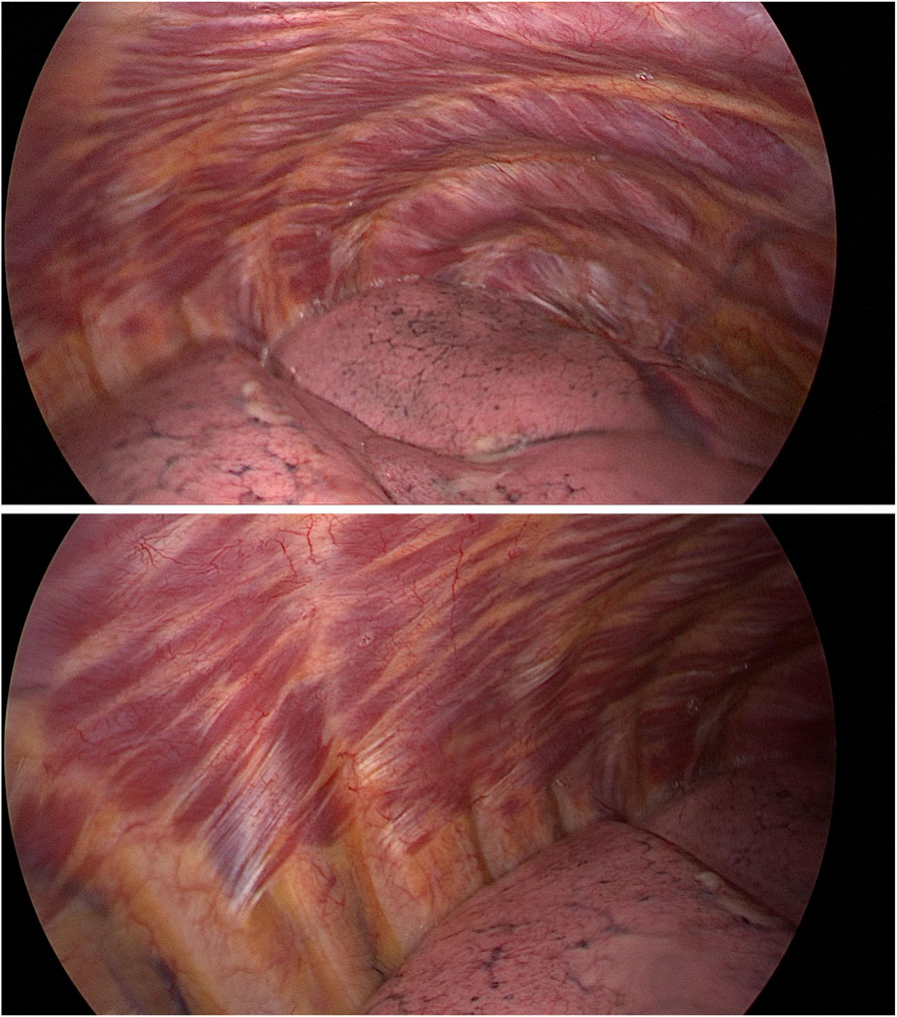
Videoscopic images of chest cavity prior to second procedure: no pleural adhesions visible

When recurrence of pneumothorax happens after pleurodesis or pleurectomy, it is often partial and attributed to incomplete scarring [18]. However, in our patient, a complete collapse of the lung at the pleurectomised side was observed with no evidence of pleural adhesions (Fig. 2), and pathological examination of the biopsy specimens showed near-normal pleura on the parietal wall. This development of a “neo-pleura” has, to our knowledge, not been described previously.

Possible risk factors for recurrence of pneumothorax after surgical intervention include residual blebs or bullae on CT scan [19], non-smokers [20], persistent air leak (PAL) after the initial drainage or pleurodesis procedure [21, 22], female sex [21], age younger than 17 years [22] and use of anti-inflammatory medication after the procedure [23].

Our patient had no visible bullae during the first intervention; however, no CT scan was performed prior to the intervention. A PAL was diagnosed after simple aspiration (complete recurrence after 36 h) and even after pleurectomy, suction had to be prolonged for a total of 10 days before sealing was achieved. The patient continued to (occasionally) smoke. As the first procedure was performed by one of the authors (TO) we can confirm that the pleurectomy was complete and that there was virtually no residual parietal pleura after the procedure. Neither the CT scan nor the endoscopic image during the second procedure showed adhesions, something which would have been likely in case of previous incomplete pleurectomy.

It is not known whether our patient had taken Non-Steroidal Anti-inflammatory Drugs (NSAIDs) for pain relief after the first operation. The standard procedure for pain relief in our hospital consists of paracetamol orally or intravenously. Daily administration of diclofenac has been shown to significantly decrease the rate of collagen deposition, fibrosis and adhesion formation in a porcine model of mechanical pleural abrasion [15]. The effects of NSAIDs on the scarring process after pleurectomy have not been studied as such, but in wound healing their negative effects have been demonstrated clearly in animal models, and have been attributed to their ability to suppress prostaglandin synthesis, a key requirement for inflammation [24, 25]. However, the detrimental effect on pleural scarring has yet to be confirmed in humans [26, 27].

Two recent reviews [28, 29] concluded that over a large cohort of patients who had benefited from a variety of combinations of procedures and followed up to 10 years afterwards, the lowest risk of recurrence was seen in the group ‘VATS wedge resection + chemical pleurodesis with talcum’ and ‘VATS wedge resection + pleural abrasion + chemical pleurodesis with talcum’. The advantage of this latter combination would be that a maximal surface can be treated and that surgical (bloody) pleurodesis is enhanced with a chemical irritative reaction. At least in the short- to medium term (2.5 years), risk of recurrence seems similar between ‘pleurectomy’ and ‘pleural abrasion + chemical pleurodesis’ [30].

## Conclusions

Our observation suggests that even a very small number of residual pleural cells may regenerate a complete pleura after VATS pleurectomy. We suggest that video assisted wedge resection, followed by pleural abrasion and chemical pleurodesis may be a more reliable choice for divers, rather than VATS pleurectomy alone.

## Data Availability

Not applicable.

## Abbreviations

PSP: Primary Spontaneous Pneumothorax
VATS: Video Assisted Thoracic Surgery
FEV1: Forced Expiratory Volume in 1 s
DLCO: Diffusion Capacity for the Lung of Carbon Monoxide
CT: Computed Tomography
PAL: Persistent Air Leak
NSAID: Non-Steroidal Anti-Inflammatory Drug

## References

[1] British Thoracic Society Fitness to Dive Group SotBTSSoCC (2003). British Thoracic Society guidelines on respiratory aspects of fitness for diving. Thorax.

[2] The medical examination and assessment of commercial divers (MA1)2015 April 20, 2018. Available from: http://www.hse.gov.uk/pubns/ma1.htm.

[3] Pneumothorax and its consequences. DAN SEAP Alert Diver. 2005 April 20, 2018. Available from: https://www.danap.org/DAN_diving_safety/DAN_Doc/pdfs/pneumothorax.pdf.

[4] Noppen M, De Keukeleire T (2008). Pneumothorax. Respiration.

[5] Treasure T (2007). Minimally invasive surgery for pneumothorax: the evidence, changing practice and current opinion. J R Soc Med.

[6] Addas RA, Shamji FM, Sundaresan SR, Villeneuve PJ, Seely AJE, Gilbert S (2016). Is VATS Bullectomy and Pleurectomy an effective method for the Management of Spontaneous Pneumothorax?. Open J Thor Surg.

[7] Chang YC, Chen CW, Huang SH, Chen JS (2006). Modified needlescopic video-assisted thoracic surgery for primary spontaneous pneumothorax : the long-term effects of apical pleurectomy versus pleural abrasion. Surg Endosc.

[8] Min X, Huang Y, Yang Y, Chen Y, Cui J, Wang C (2014). Mechanical pleurodesis does not reduce recurrence of spontaneous pneumothorax: a randomized trial. Ann Thorac Surg.

[9] Sadikot RT, Greene T, Meadows K, Arnold AG (1997). Recurrence of primary spontaneous pneumothorax. Thorax.

[10] DoD Directive (2018). AR 40–501 Standards of Medical Fitness2007 April 18.

[11] Lim MK, Peng CM, Chia KE (1985). Spontaneous pneumothorax occurring in flight. Singap Med J.

[12] Respiratory guidance material: Implementing Rules, Acceptable Means of Compliance and Guidance Material on respiratory conditions2015 April 20, 2018. Available from: http://www.caa.co.uk/Aeromedical-Examiners/Medical-standards/Pilots-(EASA)/Conditions/Respiratory/Respiratory-guidance-material-GM/.

[13] Sudduth CL, Shinnick JK, Geng Z, McCracken CE, Clifton MS, Raval MV (2017). Optimal surgical technique in spontaneous pneumothorax: a systematic review and meta-analysis. J Surg Res.

[14] MacDuff A, Arnold A, Harvey J, Group BTSPDG (2010). Management of spontaneous pneumothorax: British Thoracic society pleural disease guideline 2010. Thorax.

[15] Lardinois D, Vogt P, Yang L, Hegyi I, Baslam M, Weder W (2004). Non-steroidal anti-inflammatory drugs decrease the quality of pleurodesis after mechanical pleural abrasion. Eur J Cardiothorac Surg.

[16] Tschopp JM, Bintcliffe O, Astoul P, Canalis E, Driesen P, Janssen J (2015). ERS task force statement: diagnosis and treatment of primary spontaneous pneumothorax. Eur Respir J.

[17] West JB (2002). Why doesn’t the elephant have a pleural space?. News Physiol Sci.

[18] Cardillo G, Facciolo F, Regal M, Carbone L, Corzani F, Ricci A (2001). Recurrences following videothoracoscopic treatment of primary spontaneous pneumothorax: the role of redo-videothoracoscopy. Eur J Cardiothorac Surg.

[19] Young Choi S, Beom Park C, Wha Song S, Hwan Kim Y, Cheol Jeong S, Soo Kim K (2014). What factors predict recurrence after an initial episode of primary spontaneous pneumothorax in children?. Ann Thorac Cardiovasc Surg.

[20] Uramoto H, Shimokawa H, Tanaka F (2012). What factors predict recurrence of a spontaneous pneumothorax?. J Cardiothorac Surg.

[21] Imperatori A, Rotolo N, Spagnoletti M, Festi L, Berizzi F, Di Natale D (2015). Risk factors for postoperative recurrence of spontaneous pneumothorax treated by video-assisted thoracoscopic surgerydagger. Interact Cardiovasc Thorac Surg.

[22] Jeon HW, Kim YD, Kye YK, Kim KS (2016). Air leakage on the postoperative day: powerful factor of postoperative recurrence after thoracoscopic bullectomy. J Thorac Dis.

[23] Hunt I, Teh E, Southon R, Treasure T (2007). Using non-steroidal anti-inflammatory drugs (NSAIDs) following pleurodesis. Interact Cardiovasc Thorac Surg.

[24] Haws MJ, Kucan JO, Roth AC, Suchy H, Brown RE (1996). The effects of chronic ketorolac tromethamine (toradol) on wound healing. Ann Plast Surg.

[25] Muscara MN, McKnight W, Asfaha S, Wallace JL (2000). Wound collagen deposition in rats: effects of an NO-NSAID and a selective COX-2 inhibitor. Br J Pharmacol.

[26] Ben-Nun A, Golan N, Faibishenko I, Simansky D, Soudack M (2011). Nonsteroidal antiinflammatory medications: efficient and safe treatment following video-assisted pleurodesis for spontaneous pneumothorax. World J Surg.

[27] Lizardo RE, Langness S, Davenport KP, Kling K, Fairbanks T, Bickler SW (2015). Ketorolac does not reduce effectiveness of pleurodesis in pediatric patients with spontaneous pneumothorax. J Pediatr Surg.

[28] Elsayed HH, Hassaballa A, Ahmed T (2016). Is video-assisted thoracoscopic surgery talc pleurodesis superior to talc pleurodesis via tube thoracostomy in patients with secondary spontaneous pneumothorax?. Interact Cardiovasc Thorac Surg.

[29] Ling ZG, Wu YB, Ming MY, Cai SQ, Chen YQ (2015). The effect of pleural abrasion on the treatment of primary spontaneous pneumothorax: a systematic review of randomized controlled trials. PLoS One.

[30] Chen JS, Hsu HH, Huang PM, Kuo SW, Lin MW, Chang CC (2012). Thoracoscopic pleurodesis for primary spontaneous pneumothorax with high recurrence risk: a prospective randomized trial. Ann Surg.

